# CXCL12 gene silencing down-regulates metastatic potential via blockage of MAPK/PI3K/AP-1 signaling pathway in colon cancer

**DOI:** 10.1007/s12094-017-1821-0

**Published:** 2018-01-05

**Authors:** J. Ma, H. Su, B. Yu, T. Guo, Z. Gong, J. Qi, X. Zhao, J. Du

**Affiliations:** 1 Department of General Surgery, Gansu Provincial People’s Hospital, Lanzhou, 730000 China; 2 Department of General Surgery, The Second People’s Hospital of Lanzhou, JingYuan Road 388, Cheng Guan Ward, Lanzhou, 730000 Gansu Province China; 3 Ningxia Medical University, Yinchuan, 750000 China; 4 Traditional Chinese Medicine University of Gansu, Lanzhou, 730000 China

**Keywords:** CXCL12 siRNA, Colon cancer, Proliferation, Invasion, MAPK/PI3K/AP-1 signaling pathway

## Abstract

**Background:**

To investigate the effect of CXCL12 gene silencing on proliferation,invasion, angiogenesis and the relationship of MAPK/PI3K/AP-1 signaling pathway in colon cancer cells.

**Methods:**

RT-PCR and Western-blot were used to detect the expression of CXCL12 mRNA and protein in four colon cancer cell lines. Human colon cancer cells were transfected with CXCL12 siRNA carrying by Lipofectamine 2000. The expression of CXCL12 protein was confirmed by immunoblotting. WST-1, invasion and angiogenesis assay were used to examine the effect on proliferation, invasion and angiogenesis in colon cancer cells after CXCL12 siRNA silence, respectively. The phosphorylation of MAPK/PI3K/AP-1 protein levels was detected by Western blotting in CXCL12 siRNA suppression DLD-1 cell.

**Results:**

CXCL12 mRNA and proteins were only expressed in DLD-1 colon cancer cell lines. CXCL12 siRNA were transfected into DLD-1 cells, the expression CXCL12 proteins was significantly inhibited (*P* < 0.01), and the proliferation, invasion and angiogenesis of DLD-1 cells were inhibited significantly (*P* < 0.01). CXCL12 gene silencing resulted in blockage of MAPK, PI3K and AP-1 phosphorylation by CXCL12-induced in DLD-1 colon cancer cell.

**Conclusion:**

The silencing CXCL12 gene significantly inhibits the proliferation, invasion and angiogenesis ability of some types colon carcinoma cells through down-regulation of MAPK/PI3K/AP-1 signaling pathway.

## Introduction

Colon cancer is one of the most common human malignancies.,Its incidence in males is the third highest in the world, its incidence in females is the fourth highest in the world and its mortality rate is the third highest in the world [1]. About 40% of colon cancer patients have recurrence or metastasis within 5 years after surgery and thus the metastasis is the main reason for its poor prognosis. Therefore, exploring the fundamental mechanism of invasion, proliferation, metastasis and tumor biological behaviors at the level of cellular or molecular microenvironments are needed in clinical diagnosis and therapy.

Recent studies showed that chemokine CXCL12 and its receptor CXCR4 play an important role in the proliferation and organ-specific metastasis of colorectal cancer [2, 3]. CXCL12 (stromal derived factor-1α, SDF-1), a 68-amino-acid chemokine (8 kDa) belonging to the CXC chemokine family, is constitutively expressed in the bone marrow, skin, heart, liver and lung tissues. CXCL12 has multiple roles in tumor pathogenesis by promoting tumor growth, enhancing tumor angiogenesis, suppressing tumor immunity and participating in metastasis of carcinoma [4]. SDF-1 can specifically mediate the chemotactic movement, invasion and metastasis of breast cancer cells through CXCR4, and atypical chemokine receptor CXCR7 [5]. The combination of chemokine CXCL12 and its receptor CXCR4 can up-regulate the expressions of MMP-2 and MMP-9 in tumor cells and reduce the secretion of tissue inhibitor of metalloproteinases (TIMPs) [6]. Studies have also shown that the combination of CXCL12 and its receptor CXCR4 can activate NF-κB and increase the secretion of MMP-2 in tumor cells [7, 8]. Furthermore, expression of CXCR4 and CXCL12 predicts lymph node metastasis in colorectal [9–12], esophageal [13] and breast cancer [14]. CXCR4 has been shown to be a predictor of poor survival in nasopharyngeal carcinoma [15], renal cell carcinoma [16], gastrointestinal tumor [17].

CXCL12 siRNA was transfected into colorectal cancer cells with small interfering RNA (siRNA). The changes in biological characteristics of colon cancer cells were observed after the target gene CXCL12 was silenced, and the affected signal transduction pathways were explored to understand the molecular biological mechanisms for effect of CXCL12 on the occurrence and development of tumors and provide a new method strategy and theoretical basis for the prevention and treatment of colon cancers.

The purpose of this study was to investigate the effect of CXCL12 gene silencing on metastatic potential and the underlying mechanism in colon cancer cells. Furthermore, our study provided data to demonstrate that phosphatidylinositol MAPK/PI3K/AP-1 signaling pathway plays an important role in CXCL12 simulation and that this process is involved in the development and metastasis of colon cancer. Understanding the biologic mechanisms responsible for regulation of chemokines may enable better molecular targeted therapies to treat patients with metastatic colon cancer.

## Materials and methods

### Reagents and antibodies

Recombinant human CXCL12 was purchased from R&D Systems (Minneapolis, MN). Neutralizing monoclonal anti-human CXCL12 (anti-CXCL12 Ab), anti-human CXCR4 (anti-CXCR4 Ab) were obtained from Carbiochem (San Diego, CA, USA).

### Cell lines and culture condition

The human colon cancer cell lines HT29, CaCo-2, DLD-1 and Colo320 were obtained from American Type Culture Collection (Rockville, MD). DLD-1 and CaCo-2 were maintained in microscale essential medium eagle (Sigma Chemical Co., St. Louis, MO) with high glucose and 10% fetal bovine serum (FBS). HT-29 was cultured in McCoy’s supplemented with 10% FBS. Colo320 was maintained in RPMI-1640 medium (Sigma Chemical Co.) supplemented with 10% FBS. HUVECs were obtained from Kurabo Co. (Osaka, Japan). HUVECs were maintained in HuMedia-EG2 medium supplemented with 2% FBS, 5 ng/ml basic fibroblast growth factor, 10 μg/ml heparin, 10 ng/ml epidermal growth factor and 1 μg/ml of hydrocortisone according to the supplier’s instruction (Kurabo Co.). All cells were incubated at 37 °C in a humidified atmosphere of 5% CO_2_ in air.

### RT-PCR detect of expression of CXCL12 mRNA in colon cancer cell lines

The primers were designed using Prime 5.0 software according to the gene sequences reported by Gene Bank. The total RNA was extracted from all cell lines using the RNA OUT kit, and then the RNA concentration was determined using DNA/RNA calculator. cDNA was synthesized by reverse transcription using 5 μg of RNA, and the cDNA was used as a template for PCR amplification of CXCL12 gene. RT-PCR reaction system included 10 × RT buffer, 25 mM MgCl_2_, 0.1 MDTT, RNaseOUT and 200 U SuperScript™ III RT, which was placed in the water temperature box, respectively, at 50 °C for 50 min and at 85 °C for 5 min. 1 μl of cDNA was used for amplification reaction; the operations were carried out according to the supplier’s instructions. Primer sequence and PCR condition are shown in Table 1.

**Table 1:** Primer sequence and PCR condition

Gene name	Primer sequences	Tm (°C)	Cycles	Length (bp)	Accession number
CXCL12	F: 5′-TTCCATTTGCAAGGGAAAAG-3′	56	35	236	NM-000609
	R: 5′-ACACACAGCCAGTCAACGAG-3′				
CXCR4	F: 5′-GAAGCTGTTGGCTGAAAAGG-3′	54	35	345	NM-003467
	R: 5′-GAGTCGATGCTGATCCCAAT-3′				

### Western blotting detection of the expression of CXCL12 proteins in colon cancer cell lines

The colon cells were lysed by lysis buffer consisting of 25 mM Tris (pH 7.8) with H_3_PO, 2 mM CDTA, 10 mM DTT, 10% glycerol, 1% Triton^®^ X-100, 2 mM PMSF, 1 mM sodium orthovanadate and 10 µM leupeptin. The protein concentrations were measured by BCA protein assay kit (Pierce, Rockford, USA). 30 µg of protein samples per each lane was separated by 10% SDS–polyacrylamide gel electrophoresis and transferred to polyvinylidene membrane (Immobilo PVDF; Nihon Millipore Ltd, Tokyo, Japan). The membrane was incubated in blocking buffer for 60 min at room temperature. The blocking buffer was formed by 5% nonfat dry milk and dissolved into Tris-buffered saline which contained 0.1% Tween 20 (TBS-T). The membranes were washed by TBS-T and then immunoblotted by each diluted into 1:1000–2000 of primary antibody overnight at 4 °C. Following, the membranes were washed by TBS-T three times. The primary antibody was combined by HRP-conjugated secondary antibody in Tris-buffered saline for 60 min at room temperature. The complexes of Protein antibody were visualized with an ECL Western Blotting detection and analysis system (Amersham Biosciences, Buckinghamshire, UK). β-actin blotting served as control. The grayscale values of the strips were measured by Image J software. The relative expression level of the proteins was expressed as the ratio of the target protein to the internal reference protein.

### Design and synthesis of siRNA and its transfection into colon cancer cells

The siRNAs were designed by coding sequence of human CXCL12 gene. CXCL12 siRNA sequences: F: 5′-AUGGCUUUCGAAGAAUCGGCAUGGG-3′, R: 5′-CCCAUGCCGAUUCUUCGAAAGCCAU-3′. Four colon cancer cells were counted and incubated overnight in a 35-mm cell culture dish at 2 × 10^5^/well. Before transfection, the cells were cultured again in the fresh DMEM media containing 10% FBS and no antibiotics for 24 h. 200 pmol of Stealth™ CXCL12 siRNA or Control siRANo (the control group transfected with siRAN) was diluted with 500 μl of Opti-MEM^®^I Reduced Serum Medium; then 10 μl of Lipofectamine™ 2000 was diluted with 500 μl of Opti-MEM^®^I Reduced Serum Medium and was kept at room temperature for 5 min, and then both of them were mixed quickly and stored at room temperature for another 20 min. Thereafter, the culture cells were directly added with the mixed solution of siRNA: Lipofectamine™ 2000 at a concentration of 100 nmol/l and mixed homogeneously, and then the mixture was placed and cultured in an incubator at 37 °C. The cells were harvested at 48 h after transfection for Western blot and subsequent experiments.

### Western blot detection of the expression of CXCL12 protein in colon cancer cell line after silencing of CXCL12 gene

The 1 × 10^6^ cells/ml of each four types of colon cancer cells respectively in the untransfected group, the group transfected with CXCL12 siRNA (CXCL12 siRNA group), the group transfected with Control siRNA (Control siRNA group) was collected and lysed with Cell-Lysis buffer, the expression of CXCL12 proteins was detected using the above mentioned western blot method.

### Detection of effect of CXCL12 gene silencing on the proliferation of colorectal cancer cells (proliferation assay)

The untransfected HT-29 and DLD-1 colon cancer cells in the logarithmic phase were harvested, and each type of cells divided into the transfected group (CXCL12 siRNA): the negative control group (Control siRNA) and the untransfected group. The cells were implanted at a concentration of 1 × 10^4^ cells/100 μl in 96-well plates overnight, and the medium was exchanged. The cells were cultured for another 72 h after the culture medium was replaced; each well was added with 100 μl of CellTiter 96Aqueous One Solution Reagent, and then the plates were placed into the incubator at 37 °C for 4 h of reaction, and subsequently the microplate reader was used to measure the absorbance value at 490 nm (*D* value) for each well. Then, the absorbance value was continuously detected in each group at time points 24, 48, 72, 96 and 120 h.

### The effect of CXCL12 gene silencing on the invasion of colon cancer cells (invasion assay)

The BioCoat Matrigel Invasion Chambers (Bencton Dickinson, Bedford MA) were used to confirm invasion of colon cancer cells. Each cell was divided into the transfected group (CXCL12 siRNA), the negative control group (Control siRNA) and the untransfected group. First, cells were inoculated at density 1 × 10^5^/ml into the Martrigel pre-coated trans-wells containing polycarbonate membranes with 8-μm pores. Tran-well chambers were then placed in 24-well plates. After 24 h of incubation, the upper surfaces trans-wells were wiped by cotton swab and invaded cells were fix and stained with Diff-Quik kit. The invaded cells were counted in five microscope fields (200×). The experiment was repeated three times.

### Effect of CXCL12 siRNA on angiogenesis co-cultured with colon cancer cells in vitro

To investigate the influence of CXCL12 gene silencing on tubular formation by HUVECs, DLD-1 and HT-29 cells (transfected with CXCL12 siRNA group, Control siRNA group and untransfected group), HUVECs and fibroblasts were co-cultured using a double-chamber method in 24-well plates. DLD-1or HT-29 cells (5 × 10^4^ cells) were seeded into trans-well chambers, consisting of polycarbonate membrane with 0.45-m pores and allowed to adhere overnight. Trans-well chambers were then placed in the HUVECs/fibroblast co-culture system, and medium was exchanged every 2 days. All cells were cultured for total 11 days. The tubular formation was stained with anti-CD31 antibody by the protocols of manufacturer. The area of tubular formation was measured quantitatively over ten different fields for each condition using an image analyzer (Kurabo Co., Osaka, Japan).

### Western blot detection of changes in protein phosphorylation of PI3K/Akt/NF-B pathway after CXCL12 gene silencing

The 6 × 10^6^ cells/ml of DLD-1 cells was collected, respectively, from untransfected group, the group transfected with CXCR4 siRNA and the group transfected with Control siRNA (namely untransfected, CXCR4 siRNA and Control siRNA groups, respectively) and HT-29 cells. The media were added, respectively, with 0, 1, 10 and 100 ng/ml of CXCL12 for 15 min of stimulation, and then all cells were harvested and lysed by cell-Lysis buffer. The supernatant was collected after centrifugation. The changes in protein phosphorylation of MAPK, PI3K and AP-1 were detected by Western blot. Western blot method was previously described.

### Statistical analysis

All data are presented as means standard deviations (SD). Differences in the mean of two groups were analyzed by an unpaired *t* test. Multiple group comparison was performed by one-way ANOVA with a post hoc test for subsequent individual group comparisons. *P* < 0.05 was considered statistically significant. Mean values and SD were calculated for experiments performed in triplicate (or more).

## Results

### The expressions of CXCL12, CXCR4 mRNA and proteins in colon cancer cell lines

**Fig. 1:**
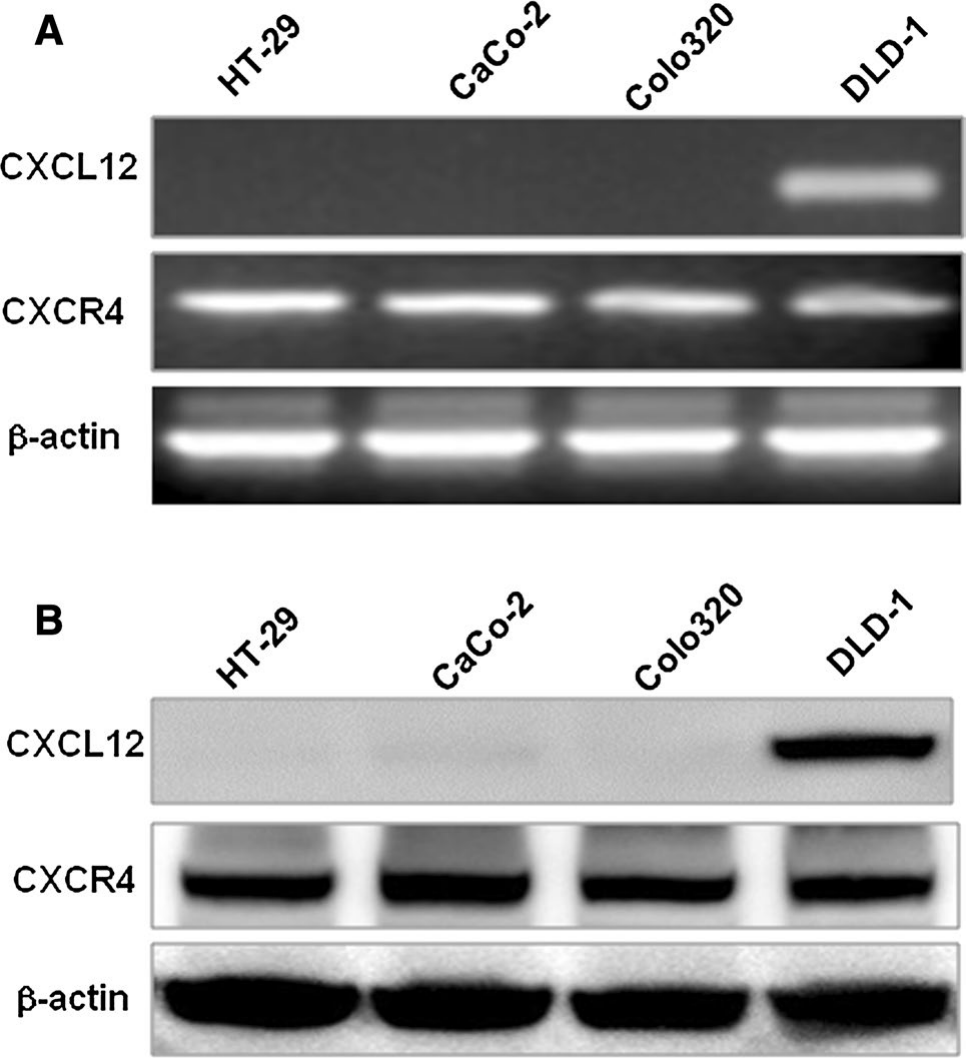
Expression of CXCL12 and CXCR4 in colon cancer cell lines. **a** Detection of CXCL12 and CXCR4 mRNA in colon cancer cells. PCR products stained with ethidium bromide were subjected to 1.5% agarose gel electrophoresis. **b** The protein expression of CXCL12 and CXCR4 in colon cancer cell lines was confirmed by Western blotting analysis. β-Actin served on a loading control

CXCL12 mRNA was expressed only in DLD-1 colon cancer cell line, while CXCR4 mRNA was expressed in four colon cancer cell lines. β-actin was taken as a positive control (as shown in Fig. 1a). The experimental results were the same with those of RT-PCR. Western blot showed that CXCL12 protein was only expressed in DLD-1 cells, and CXCR4 was expressed in four colon cancer cell lines. β-actin was taken as the positive control (as shown in Fig. 1b).

### Effect of CXCL12 siRNA transfection on secretion of CXCL12 proteins in colon cancer cells

**Fig. 2:**
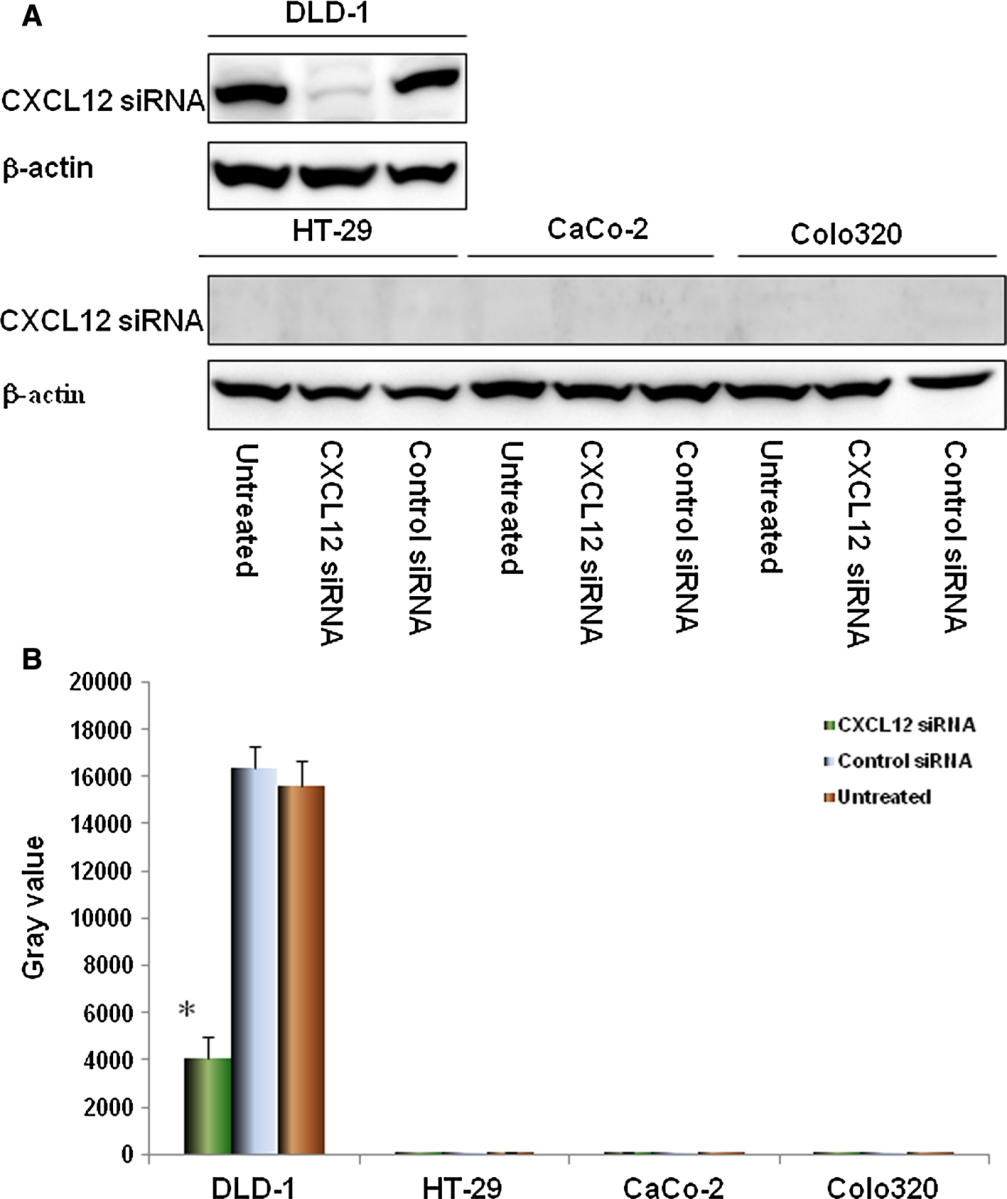
The expression of CXCL12 protein in colon cancer cell line after silencing of CXCL12 gene. Knockdown of CXCL12 by CXCL12 siRNA was confirmed by immunoblotting (**a**) in all four colon cancer cell lines. siRNA duplex oligoribonucleotides were transfected into cells for 48 h; the total RNA and proteins were extracted and then western blot. The grayscale values of the strips were measured by Image J software (**b**). Multiple comparisons were performed by one-way ANOVA followed by SNK test. Values are expressed as mean ± SD. Bars indicated SD

DLD-1, HT-29, CaCo-2 and Colo320 cells were transfected with siRNA that specifically targets CXCL12 gene, the expressions of CXCL12 proteins was detected by Western blot. The experimental results showed that: after CXCL12 gene silencing, compared with the untransfected and control siRNA groups and positive control β-actin (shown in Fig. 2a), the expressions of CXCL12 proteins in four colon cancer cells were significantly inhibited (*P* < 0.01, respectively, compared with the untransfected and control siRNA groups), and the experiment showed that CXCL12 siRNA primer design and cell transfection were successful (as shown in Fig. 2b).

### Effect of CXCL12 siRNA on proliferation of colon cancer cells

**Fig. 3:**
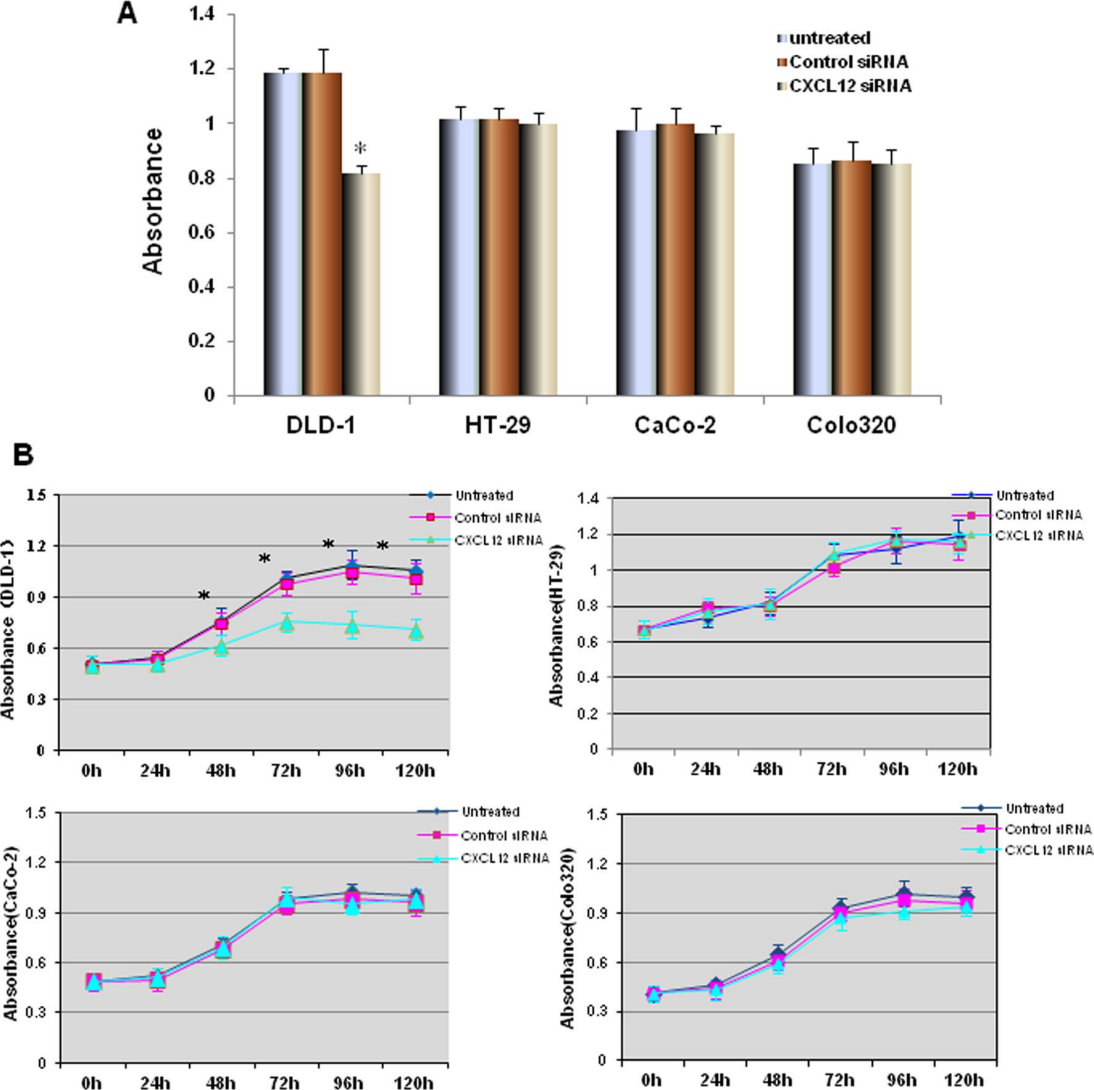
The effect of CXCL12 gene silencing on the proliferation of colon cancer cells. **a** DLD-1, HT-29 CaCo-2 and Colo320 cells were transfected by CXCL12 siRNA and Control siRNA for 24 h; the proliferation of cancer cells was measured by WST-1 assay. The proliferation of DLD-1 cells in CXCL12 siRNA group was significantly inhibited (compared with the untransfected and control siRNA groups, *P* < 0.01, respectively). **b** The cell proliferation curve showed that there was no significant difference in the proliferation of DLD-1 colon cancer cells before 24 h; the proliferation of CXCL12 siRNA group was significantly lower than those in the untransfected and control siRNA groups after 48, 72, 96, 120 h (*P* < 0.01). Multiple comparisons used the method of one-way ANOVA and followed by the SNK test. Values are expressed as mean ± SD. Bars indicated SD, *P < 0.01

After being transfected with CXCL12 siRNA and Control siRNA for 24 h, HT-29 and DLD-1 colon cancer cells were cultured for 72 h, and the proliferation of cancer cells was measured by WST-1 assay. The results showed that after CXCL12 gene silencing, the proliferation of DLD-1 cells in CXCL12 siRNA group was significantly inhibited (compared with the untransfected and control siRNA groups, *P* < 0.01, respectively, as shown in Fig. 3a). The cell proliferation curve showed that there was no significant difference in the proliferation of DLD-1 colon cancer cells before 24 h; the proliferation of colon cancer cells in CXCL12 siRNA group was significantly lower than those in the untransfected and control siRNA groups after 48, 72, 96, 120 h (*P* < 0.01, compared with the untransfected and control siRNA groups). HT-29, CaCo-2 and Colo320 cells transfected with CXCL12 siRNA there were no significant change (compared with the untransfected and control siRNA groups respectively, Fig. 3b).

### Effect of CXCL12 siRNA on invasion of colon cancer cells

**Fig. 4:**
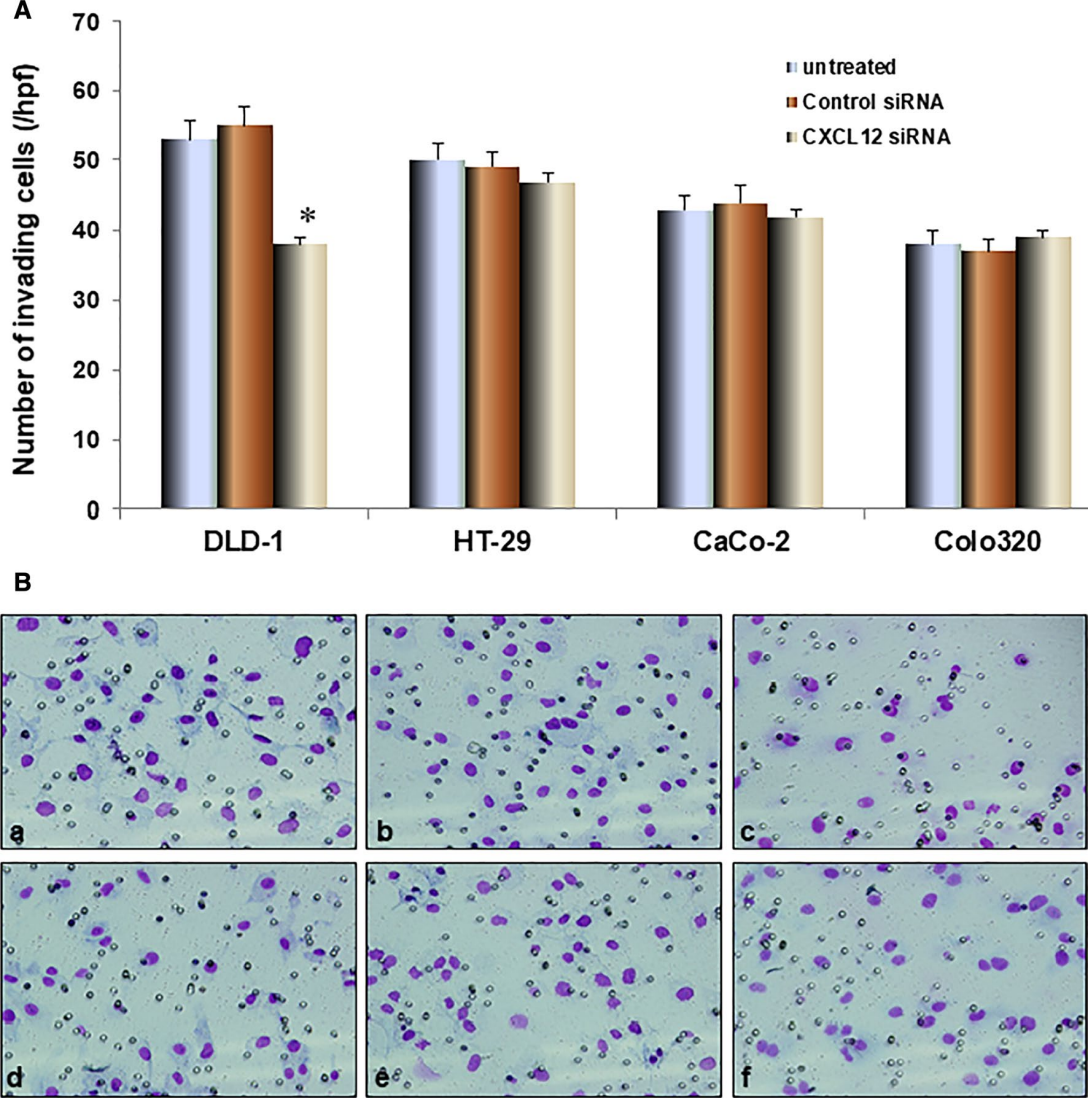
The effect of CXCL12 gene silencing on the invasive ability of colon cancer cells. **A** DLD-1 and HT-29 invasion by influence of CXCL12 siRNA was assessed by the BD Bio-Coat Matrigel invasion assay. The cells were incubated for 24 h; the invading cells were fixed and stained with Diff-Quick stain. The invading cells were counted in five random microscopic fields (×200). The invasive ability of DLD-1 transfected with CXCL12 siRNA group was significantly decreased compared with the untransfected and control siRNA groups (*P* < 0.01). The invasive ability of HT-29 that transfected CXCL12 siRNA group had no significant change compared with the untransfected and control siRNA groups. **B** The invasive figure is as follows: *a* DLD-1 cells untreated; *b* DLD-1 cells Control siRNA; *c* DLD-1 cells CXCL12 siRNA; *d* HT-29 cells untreated; *e* HT-29 cells Control siRNA; *f* HT-29 cells CXCL2 siRNA. Multiple comparisons used the method of one-way ANOVA and followed by the SNK test. Columns, relative invading number. Bars indicate SD, *P < 0.01

The invasion experiment results showed that the invasion ability of DLD-1 of CXCL12 siRNA group was significantly reduced compared with the untransfected and control siRNA groups (*P* < 0.01, as shown in Fig. 4). The invasion ability of colon cancer cell HT-29 of CXCL12 siRNA group had no significant change compared with the untransfected and control siRNA groups.

### Effect of colon cancer cells with CXCL12 gene silencing on tube formation

**Fig. 5:**
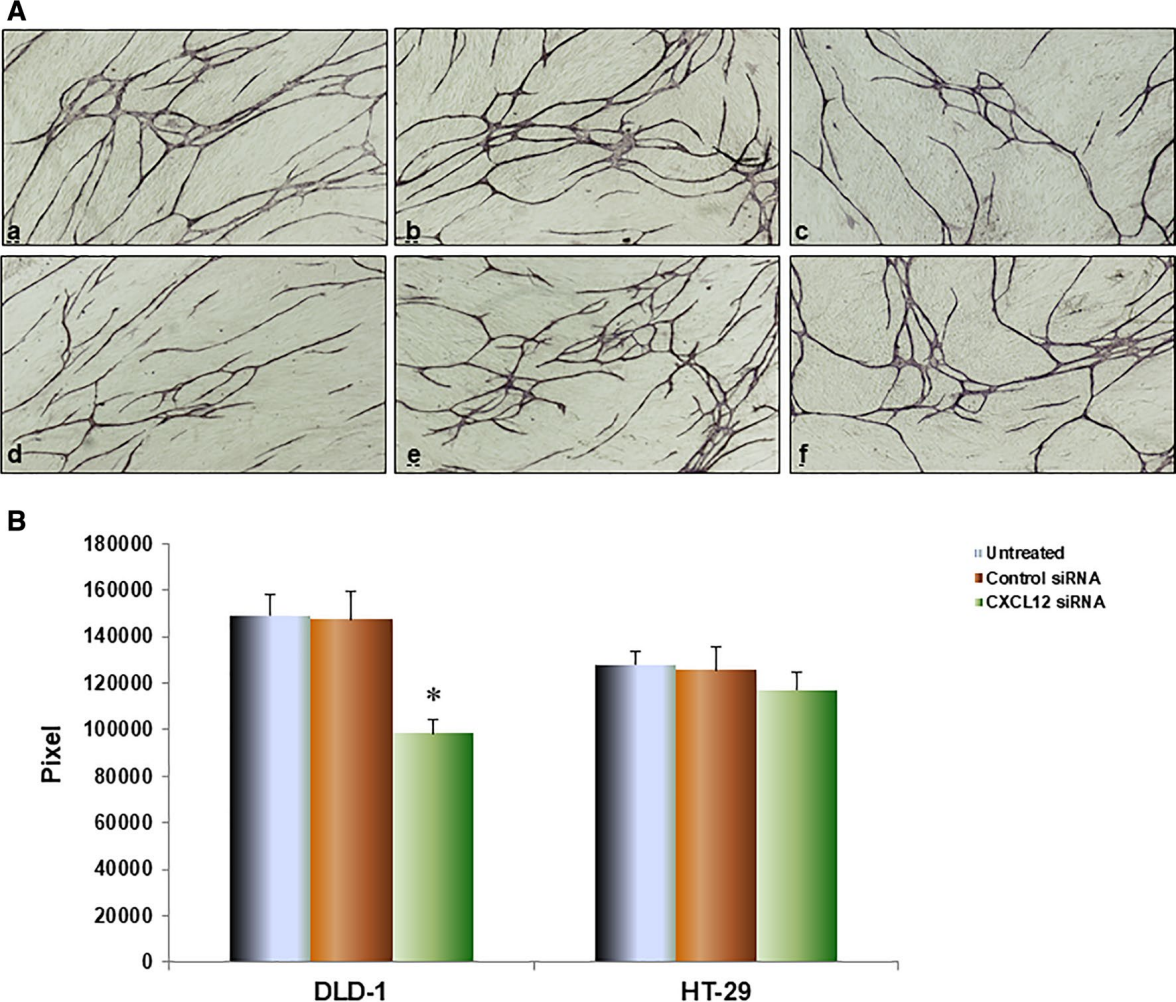
CXCL12 gene silencing influence on tube formation by HUVEC. Colon cancer cells were co-cultured with HUVEC and fibroblast using double-chamber method. All cells were cultured for total 11 days. The tubular formation was stained with anti-CD31 antibody by the protocols of manufacturer. The area of tubular formation was measured quantitatively over ten different fields for each condition using an image analyzer. **A** The image of angiogenesis shows *a* DLD-1 cells untreated; *b* DLD-1 cells Control siRNA; *c* DLD-1 cells CXCL2 siRNA; *d* HT-29 cells untreated; *e* HT-29 cells Control siRNA; *f* HT-29 cells CXCL12 siRNA. **B **The tubular formation of HUVEC was significantly inhibited by co-culture with CXCL12 siRNA DLD-1 cells compared with untransfected and control siRNA groups, respectively (*P* < 0.01). HT-29 of CXCL12 siRNA group had no significant change compared with the untransfected and control siRNA groups. Multiple comparisons used the method of one-way ANOVA and followed by the SNK test. Bars indicate SD, *P* < 0.01

To further investigate CXCL12 gene silencing influence on tube formation by HUVEC, we co-cultured with colon cancer cell and HUVEC + fibroblast using double chamber methods to determine the interaction among them. The tubular formation of HUVEC was significantly inhibited by co-culture with CXCL12 siRNA DLD-1 cells compared with untransfected and control siRNA groups, respectively (*P* < 0.01, Fig. 5). HT-29 of CXCL12 siRNA group had no significant change compared with the untransfected and control siRNA groups.

### Effects of CXCL12 siRNA on phosphorylation of major proteins in MAPK/PI3K/AP-1 signaling pathway

**Fig. 6:**
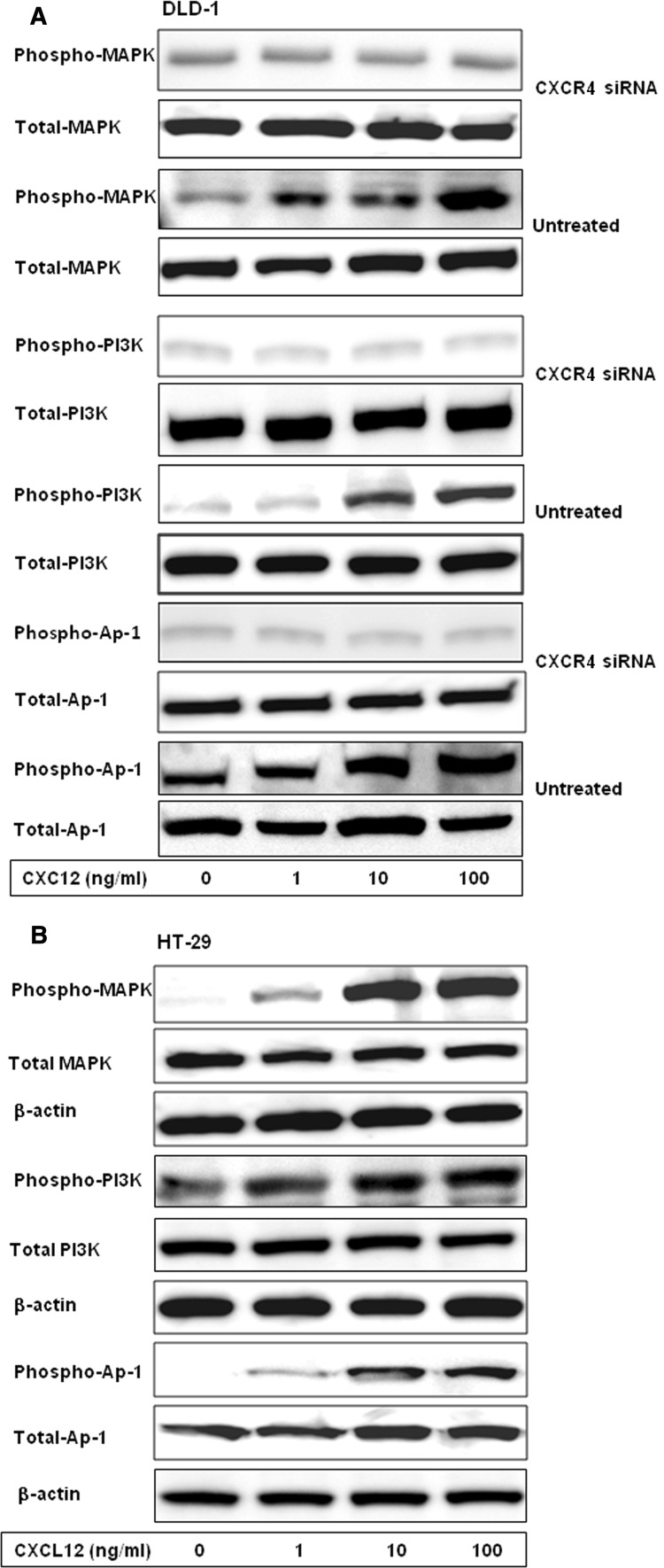
Effects of CXCR4 siRNA on phosphorylation of major proteins in MAPK/PI3K/AP-1 signaling pathway. Colon cancer cells were stimulated by different concentrations of CXCL12 for 15 min. The proteins were extracted and separated by SDS-PAGE, transferred to membranes, and the membranes probed with antibody directed against phospho-MAPK, phospho-PI3K, phospho-AP-1, total MAPK, total PI3K and total AP-1. After different concentrations of CXCL12 were used to stimulate the DLD-1 CXCR4 siRNA, untransfected, control siRNA groups and HT-29 cells for 15 min, the effects of CXCL12 on phosphorylation of member proteins in MAPK/PI3K/AP-1 signaling pathway were detected by Western blot. **a **The phosphorylation levels of MAPK, PI3K and AP-1 proteins in CXCR4 siRNA group after being simulated by different concentrations of CXCL12 were significantly weaker than the untransfected and control siRNA groups. **b** The phosphorylation of MAPK, PI3K and AP-1 proteins were positively correlated with the concentration of CXCL12 in HT-29 cells

After different concentrations of CXCL12 (0, 1, 10 and 100 ng/ml) were used to stimulate the CXCR4 siRNA transfected, untransfected and control siRNA, and HT-29 for 15 min, the effects of CXCL12 on phosphorylation of member proteins in MAPK/PI3K/AP-1 signaling pathway were detected by Western blot. The results showed that the phosphorylation of MAPK, PI3K and AP-1 proteins were positively correlated with the concentration of CXCL12 in DLD-1 control siRNA, untreated groups and HT-29. The phosphorylation levels of MAPK, PI3K and AP-1 proteins in the CXCL12 siRNA group after being simulated by different concentration of CXCL12 were significantly weaker than those of the untransfected and control siRNA groups (Fig. 6).

## Discussion

The metastasis of colon cancer is a complex and non-random multi-stage process involving critical steps such as proliferation, movement, migration, adhesion, invasion, growth, homing, immune escape and neovascularization of tumor cells. The specificity of target organ and the way for circulating tumor cell chemotaxis towards target organ are the key to reveal the mechanism of colon cancer metastasis. It has been demonstrated that chemokines and their receptors are involved in distant metastases of tumors and have characteristics similar to those of leukocyte chemotaxis [13]. As key signaling molecules in tumor microenvironment, the chemokines play an important role in invasion and metastasis of carcinoma. As an important component of the chemokine family, the fibroblast-derived CXCL12 has been shown to play an important role in the development of various cancers such as breast cancer, lung cancer and thyroid cancer. CXCL12 and its specific receptors CXCR4 have been shown to be associated with the growth and metastasis of a variety of malignant tumors [14–18]. The other studies have shown that the expressions of CXCL12 and CXCR4 in colon cancer patients are associated with liver metastasis, recurrence rate and survival rate in colorectal cancer patients [19]. While the inhibition of CXCL12 and CXCR4 can significantly reduce tumor cell proliferation and metastasis [20, 21], indicating that CXCL12 is closely related to development, outcome and prognosis of colon cancers.

The target gene silencing method of RNA interference technology has been widely used in clinics, and the cells in division phase or in intermission phase can be suppressed by RNA interference, with less toxic side effects. Therefore, RNA interference has become one of vital tools for the basic research and gene treatment [22, 23]. Based on the above, this study conducted a series of experimental studies through in vitro transfection of CXCL12 siRNA. Our results revealed that CXCL12 was only expressed in DLD-1, while CXCR4 was expressed in all cell lines. Western blot results showed that DLD-1cells transfected with CXCL12 siRNA inhibited the expression of CXCL12 proteins, indicating that the effect of silencing target gene was significantly, this result provided a basis for detecting changes in the biological activity of colon cancer cells. The proliferation and invasion assay confirmed that proliferation and invasion ability of DLD-1 colon cancer cells in the group transfected with CXCL12 siRNA were significantly inhibited compared with control siRNA group and untreated group, this indicated that CXCL12 siRNA could inhibit the proliferation (Fig. 3) and invasion (Fig. 4) of colon cancer cells with expression CXCL12, but there is no influence for HT-29, CaC0-2 and Colo320 cell, because they have not secretion of CXCL12.

To further investigate CXCL12 gene silencing influence on tube formation by HUVEC, we co-cultured with colon cancer cell and HUVEC/fibroblast using double-chamber methods to determine the interaction among them. We aimed to explore the influence of different secreted CXCL2 from colon cancer cells on tube formation by HUVECs. The tubular formation of HUVEC was significantly inhibited by co-culture with CXCL12 siRNA DLD-1 cells compared with untransfected and control siRNA groups, respectively (Fig. 5). HT-29 of CXCL12 siRNA group had no significant change compared with the untransfected and control siRNA groups. This is consistent with the results of a related study which showed that CXCL12 can enhance the metastases of colon cancer [2]. Our results suggest that specific inhibition of CXCL12 may be a convenient and effective way to treat expression CXCL12 of colon cancer patients with expression of CXCL12.

In addition, the relationships of phosphorylation of components of MAPK/PI3K/Ap-1 signaling pathway with CXCL12 were examined in this study, and the correlative mechanism for the inhibitive effect of CXCL12 siRNA on the proliferation and invasion of colon cancer cells was investigated. The results showed that phosphorylations of MAPK, PI3K and AP-1 were closely related to CXCL12, and MAPK/PI3K/AP-1 signaling pathway was activated gradually with an increasing concentration. This result indicates that CXCL12 enhances the proliferation and invasion of colon cancer cells through the MAPK/PI3K/AP-1 signaling pathway. After transfection of CXCL12 siRNA, the phosphorylation levels of components of MAPK/PI3K/AP-1 pathway are significantly inhibited by the silencing of CXCL12 gene, which indicates that CXCL12 siRNA can effectively bind to its target gene in colorectal cancer cells, inhibit its translation and expression and then inhibit the phosphorylation of the main members of the downstream pathway to inhibit the signal transduction pathway and reverse the phosphorylation cascade reaction between the member proteins of the MAPK/PI3K/AP-1 pathway to reduce the proliferation and invasion abilities of colon cells. In addition, the accumulated evidence suggests that different combinations of AP-1 dimers with different biological functions, which have been proposed in the antagonistic regulation of peroxisome proliferator-activated receptor *γ*, can affect the obesity and liver function [24–26]. The cell type and contextual signals may alter the AP-1 dimer composition, thereby determining the final cell fate. Therefore, MAPK/PI3K/AP-1 signaling pathway has become a hot topic in tumor research and also provides a specific target spot for cancer treatment [27–29].

In conclusion, the CXCL12 gene silencing can significantly inhibit the metastatic potential of colon cancer by siRNA interference, and its mechanism may be related to CXCL12 siRNA down-regulate the cascade of MAPK/PI3K/AP-1 pathway. However, in CXCL12 siRNA-mediated inhibitive effect on proliferation, invasion and angiogenesis of colon cancer cells, the discovery of MAPK/PI3K/AP-1 pathway downstream target needs to be analyzed by more precise experiments; the inhibition of the cascade transmission between member proteins of MAPK/PI3K/AP-1 pathway and the interaction between the target gene and the target pathway of CXCL12 will also be the next research directions of this research group, and clarifying these mechanisms will help to enable the chemokine-mediated gene targeting to be used in the clinical treatment of colon cancers as a new therapeutic technique.

## Abbreviations

CXCL12: Stromal cell-derived factor-1
CXCR4: CXC chemokine receptor 4
HUVEC: Human umbilical vein endothelial cell
FB: Fibroblast
ELISA: Enzyme-linked immunosorbent assay
PI3K: Phosphatidylinositol 3-kinase
Akt: Protein kinase B
NF-κB: Nuclear transcription factor kappa-B
MAPK: Mitogen-activated protein kinase
AP-1: Activator protein 1
